# Hyperlocal Air Pollution Mapping: A Scalable Transfer Learning LUR Approach for Mobile Monitoring

**DOI:** 10.1021/acs.est.4c06144

**Published:** 2024-07-31

**Authors:** Zhendong Yuan, Jules Kerckhoffs, Hao Li, Jibran Khan, Gerard Hoek, Roel Vermeulen

**Affiliations:** † Institute for Risk Assessment Sciences, Utrecht University, 3584 CM Utrecht, Netherlands; ‡ Professorship of Big Geospatial Data Management, Technical University of Munich, 85521 Ottobrunn, Germany; § Department of Environmental Science, Aarhus University, DK-4000 Roskilde, Denmark; ∥ Danish Big Data Centre for Environment and Health (BERTHA), Aarhus University, DK-4000 Roskilde, Denmark; ⊥ Julius Centre for Health Sciences and Primary Care, University Medical Centre, Utrecht University, 3584 CX Utrecht, The Netherlands

**Keywords:** air pollution, ultra fine particles (UFP), geographic principles, unsupervised transfer learning, land use regression model (LUR), inverse distance-weighted model (IDW), domain shift

## Abstract

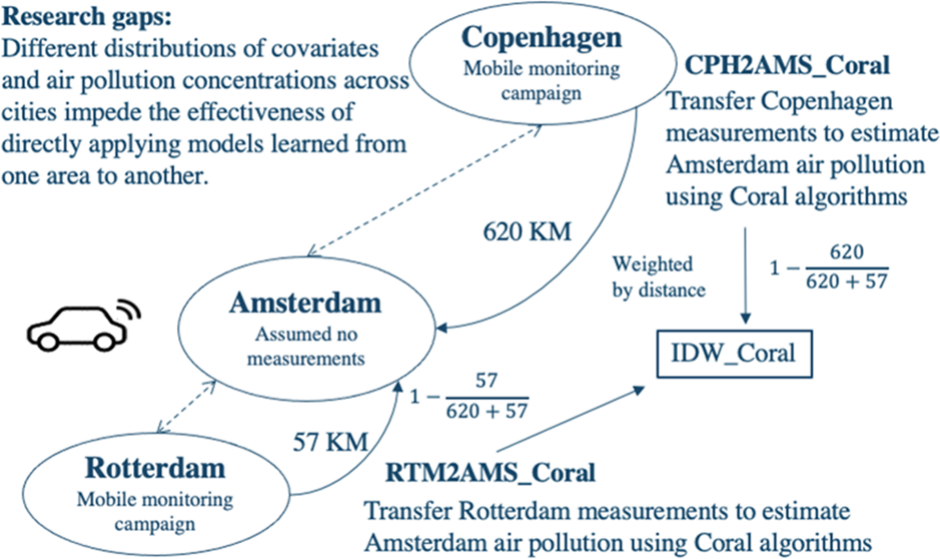

Addressing the challenge of mapping hyperlocal air pollution in areas without local monitoring, we evaluated unsupervised transfer learning-based land-use regression (LUR) models developed using mobile monitoring data from other cities: CORrelation ALignment (Coral) and its inverse distance-weighted modification (IDW_Coral). These models mitigated domain shifts and transferred patterns learned from mobile air quality monitoring campaigns in Copenhagen and Rotterdam to estimate annual average air pollution levels in Amsterdam (50m road segments) without involving any Amsterdam measurements in model development. For nitrogen dioxide (NO_2_), IDW_Coral outperformed Copenhagen and Rotterdam LUR models directly applied to Amsterdam, achieving MAE (4.47 μg/m^3^) and RMSE (5.36 μg/m^3^) comparable to a locally fitted LUR model (AMS_SLR) developed using Amsterdam mobile measurements collected for 160 days. IDW_Coral yielded an *R*^2^ of 0.35, similar to that of the AMS_SLR based on 20 collection days, suggesting a minimum requirement of 20-day mobile monitoring to capture city-specific insights. For ultrafine particles (UFP), IDW_Coral’s citywide predictions strongly correlated with previously published mixed-effect models fitted with 160-day Amsterdam measurements (Pearson correlation of 0.71 for UFP and 0.72 for NO_2_). IDW_Coral demands no direct measurements in the target area, showcasing its potential for large-scale applications and offering significant economic efficiencies in executing mobile monitoring campaigns.

## Abstract 2

Inverse Distance-Weighted CORrelation ALignment (IDW-Coral) leverages existing mobile monitoring data to cost-effectively estimate hyperlocal air pollution in European urban areas, without requiring local measurements.

## 1 Introduction

Mobile monitoring campaigns have proven highly effective in capturing hyperlocal variations (e.g., on 50m road segments) of regulated and unregulated air pollution.^1−4^ However, a city-wide mobile monitoring campaign is time-consuming and labor-intensive. The accuracy of mapping long-term (e.g., annual) concentrations using mobile measurements relies on the frequency of revisits per location, which necessitates an extended collection duration, especially when covering a large geographic area. An open scientific question is how to efficiently scale up mobile monitoring campaigns to cover larger spatial areas.

Remote sensing products have a large spatial coverage, but their resolutions are often very coarse such as 7 km × 3.5 km (TROPOMI)^5^ or 13 km × 24 km (OMI)^6^ for nitrogen dioxide (NO_2_). Additionally, satellite observations do not cover all pollutants such as ultrafine particles (UFP). To preserve the hyperlocal variations, previous studies scale up the application of mobile measurements by exploring the generalization of Land-Use Regression (LUR) models. These studies trained a LUR model using the mobile measurements from one already collected area (the source area). Then, they directly applied this trained model to estimate air pollution concentrations in another city (the target area).^10^ This approach ignores the potential differences in emission patterns between the source and target areas, often resulting in uncertainty and instability in performance. For linear regression-based LUR models, an alternative is to fix the model structure, while only recalibrating the coefficients based on the city-specific data. Previous studies showed better performance than directly applied LUR models without calibration for nitrogen dioxide (NO_2_),^7^ ultrafine particles (UFP),^8^ and Particle Number Concentration (PNC).^9,10^ However, the local measurements that can be used for recalibration are often scarce or unavailable in many areas.

Transfer learning methods are designed to incorporate the domain discrepancy between source and target areas. These methods are known for their ability to enhance training by leveraging knowledge obtained from another model trained on a similar task. Our previous papers applied supervised transfer learning algorithms to transfer the short-term mobile measurements to predict long-term air pollution concentrations by taking fixed-site measurements as the target labels.^11,12^ Target labels refer to the air pollution measurements in the target area. The supervised model is trained to establish a mapping from covariates to these target labels. However, encountering the common constraint of no target labels (i.e., no local measurements in the target area), we propose the use of unsupervised transfer learning methods. This approach aims to mitigate the domain difference by harmonizing the feature space, holding the potential to ensure model performance during the transfer process. Unsupervised transfer learning methods are broadly applied in tasks such as deep-learning-based building detection from satellite images^13^ and regression tasks involving unbalanced sampling.^14^ However, to our knowledge, their applications in air pollution modeling have yet to be explored.

This study aims to estimate hyperlocal air pollution maps for areas without local measurements. We evaluated the transferability of an unsupervised transfer learning algorithm – CORrelation ALignment (Coral) which can transfer knowledge from a single source area to the target area, requiring no measurements in the target area. Further adapted from Coral, we developed an IDW-based framework to assemble individual Coral models (i.e., IDW_Coral). It leverages the fundamental geographic principles (Tobler’s first law of geography) to fuse knowledge learned from multiple mobile-monitored areas. We applied these transfer learning LUR models to transfer mobile measurements collected from Copenhagen and Rotterdam to estimate the air pollution concentrations in Amsterdam in fine-scaled spatial resolution. Without including Amsterdam measurements, our investigation focuses on whether our proposed transfer learning LUR models can achieve comparable performance to a local LUR model trained on local mobile measurements.

## 2 Method and Data

We utilized mobile monitor data from Copenhagen and Rotterdam as the source area and Amsterdam as the target area. There is no spatial overlap between the three cities. The proposed Coral and IDW_Coral models were compared with (1) the local reference LUR models (AMS_SLR), developed using Amsterdam mobile measurements with sequentially increasing collection days (1 to 160); and (2) the directly applied LUR models, which were trained solely on Copenhagen and Rotterdam data and then directly applied, without parameter re-estimation, to estimate Amsterdam air pollution levels. Model performance was evaluated by 82 external long-term fixed-site monitors for NO_2_ (out-of-sample validation). Additionally, due to the absence of external fixed-site validations of UFP, model predictions of NO_2_ and UFP were compared to our previously published mixed-effect models trained using all Amsterdam mobile measurements of NO_2_ and UFP.^1,3^

### 2.1 Data Collection

Our mobile monitoring campaign in Amsterdam was conducted for 10 months, from May 2019 to Feb 2020, on weekdays (160 days), mainly between 9:00 and 20:00. The campaign measured each road multiple times on separate days to measure air pollution concentrations repeatedly. One Hz NO_2_ was measured by CAPS, Aerodyne Research Inc., Massachusetts, and 1 Hz UFP was measured using EPC 3783, TSI Inc. Minnesota. The raw mobile data consisted of GPS points paired with air pollution measurements and underwent preprocessing as described previously.^1^ The preprocessing included removing unrealistic 1-s values (remove those below 0 or above 500 μg/m^3^ and below 250 or above 500,000 particles/cm^3^ for NO_2_ and UFP, respectively), employing percentiles for winsorizing (set values above the 97.5th percentile to the value of the 97.5th percentile and values below 2.5th percentile to the value of the 2.5th percentile) and temporal correcting data using a reference site (only for NO_2_ and not for UFP due to the absence of a routine reference site). Afterward, the raw data was snapped to the nearby 50m road segments (referred to as the target measurements, used only to develop the reference model). We first computed the mean of GPS-based measurements for each road segment per drive day. Then the mean of these average values of all drive days was used as mobile measurements (i.e., “mean of means”^1^). Table 1 summarizes the basic statistics of the data used.

**Table 1:** Summary of the Source, Target, and Validation Data

			**NO**_**2**_			**UFP**		
**data set**	**data source**	**time frame**	measured road segments	# drive pass^a^ (mean)	mean concentrations (μg/m^3^)	measured road segments	# drive pass^a^ (mean)	mean concentrations (particles/cm^3^)
Amsterdam mobile data	AirView mobile campaign	10 months 160 collection days 2019-05-20 to 2020-02-27	47,670	6.8	28.6	47,327	5.8	32,822
Copenhagen mobile data	AirView mobile campaign	30 collection days 2019-02-11 to 2019-03-26	13,736	1.7	22.4	20,495	1.9	15,186
Rotterdam mobile data	Ri-Urban project	30 collection days 2022-11-16 to 2022-12-22	59,269	1.8	23.5	61,272	1.8	23,172
external fixed-site validation data (Palmes)	GGD^c^	10 months 160 collection days May 2019 to March 2020, Amsterdam	82 sites	N.A.^b^	28.0	not available		
model comparison	Google insights^d^	Amsterdam predictions from mixed-effect model based on 160 collection days	47,962	N.A.^b^	28.8	47,962	N.A.^b^	22,175

a Drive pass is defined as the number of different dates of drive passing.

b N.A.: not applicable.

c GGD: Amsterdam Municipal Health Service.

d Public access via: https://insights.sustainability.google/labs/airquality

The source data for the transfer learning models was obtained from mobile campaigns conducted in Copenhagen (AirView project 2019)^3,11,12^ and Rotterdam (Ri-Urbans project 2022).^15,16^ The collection schema and instrumentation are designed following the Amsterdam mobile campaign mentioned above (weekdays only). We used the measurements collected during the initial 30 days as an example of feasible short-term surveys in multiple cities. Data collected between February 11, 2019, and March 26, 2019, was included in the Copenhagen campaign. For Rotterdam, the data covered November 16, 2022, to December 22, 2022. Following the same preprocessing methods, extreme values were excluded, and the data was aggregated and snapped to 50-m road segments.

All predictor variables were identical for all models. The predictor variables are aligned with those used in our previous papers.^1,11^ The predictor features used were: (1) land use extracted from the Copernicus CORINE data set, which is a harmonized pan-European land use data set;^17^ (2) traffic information such as traffic counts and road types derived from the Dutch national road network (NWB);^18^ and (3) population density downloaded from Central Bureau of Statistics Netherlands (CBS).^19^ The specific variables are listed in Table 2 and the details including evaluated buffer sizes are summarized in Appendix Table S1. Regarding the variations in predictor variables throughout the year, variables such as land use and land cover and population density exhibit minimal fluctuations. Traffic intensity is represented by the mean of the annual volume, aligning with our objective to estimate the annual mean of air pollution concentrations.

**Table 2:** List of Predictor Features

**category**	**predictor feature**
**land use**^a^	agricultural land area; airport area; industry area; natural and forested areas; port area; residential land area; transportation area; urban green area; water area
**traffic**^b^	traffic intensity on nearest road; traffic intensity on nearest major road; heavy-duty traffic intensity on nearest road; heavy-duty traffic intensity on nearest major road; road length of all roads; road length of all major roads; traffic intensity on all roads; traffic intensity on all major roads; heavy-duty traffic intensity on all roads; heavy-duty traffic intensity on major roads
**population**^a^	population density

a With buffers of 100, 300, 500, 1000, 5000 m.

b With buffers of 25, 50, 100, 300, 500, 1000 m.

To assess the accuracy of long-term air pollution predictions, we used measurements from 82 Palmes tube monitoring sites deployed by Amsterdam Municipal Health Service (GGD) as the NO_2_ validation data.^20^ The Palmes tubes data consisted of repeated 4-weekly measurements throughout the year, covering all AMS and its surroundings. We aligned Palmes monitoring data with the same period as our Amsterdam mobile campaign and selected measurements within 20m of the nearest road segment. The most common accuracy metrics such as the squared Pearson correlation (*R*^2^), mean absolute error (MAE), and root-mean-square error (RMSE) were used to assess model performance.

No external monitoring sites were available for UFP in Amsterdam. UFP is not routinely monitored in The Netherlands and Denmark. To assess the accuracy of UFP predictions, we compared the predictions of IDW_Coral with our previously published mixed-effect model that was trained using all Amsterdam mobile data (10 months, 160 collection days). This method has been demonstrated as an efficient and accurate approach in our previous papers^1,3^ and the model estimations can be publicly accessed in the Google Insights Explorer.^21^ To align with UFP, the NO_2_ predictions of IDW_Coral were also compared to our previously published NO_2_ mixed-effect model. In addition to MAE and RMSE, the Pearson correlation (r) and Concordance Correlation Coefficient (CCC)^22^ were used to quantify the correlation and accuracy of the agreement to the mixed-effect model. Pearson correlation primarily measures the strength and direction of a linear relationship between two variables. Meanwhile, CCC evaluates both the correlation and accuracy of the agreement (overall agreement) between two sets of variables. It is more robust to outliers and does not assume linearity.

### 2.2 Transfer Learning LUR Models

#### 2.2.1 Coral

CORrelation ALignment (Coral) is an unsupervised feature-based transfer learning method. It aligns the covariance of the input features of the source and target domains. Coral transforms source features to minimize the difference between the covariance matrix of the input target data and the one of the transformed input source data. The source features transformation is described by the following optimization problem, eq 1.^23^ Specifically, it is a “two-stage” method. In the first stage, Coral conducts feature alignment on source data, decoding the input feature space. Subsequently, in the second stage, Coral fits a regressor, such as ridge regression, on the transformed target domain data within the encoded feature space, utilizing the knowledge gained from the source domain. This allows the model to generalize better to the target domain and requires no target measurements. We implemented CPH2AMS_Coral and RTM2AMS_Coral by transferring the knowledge from Copenhagen and Rotterdam respectively to estimate air pollution in Amsterdam using the Python package “*ADAPT*”.^24^
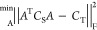
1Where *C*_S_ and *C*_T_ are the covariance matrices. *A* is the applied transformation. ∥*∥_F_^2^ denotes the squared matrix Frobenius norm.

The data set used to develop models must be distributed similarly to the target population for many statistical applications. The same principle also applies to LUR models. The training (source) data must represent the target data on which the model is applied later. This representativeness can be mathematically formulated: *P*_source_(*X,Y*) = *P*_target_(*X,Y*). Where *X* represents the predictor variables (e.g., land use, population, traffic intensity), *Y* is the response variable (air pollution measurements) and *P* is the possibility function.

When training in one area (source) and applying the fitted model to make predictions in the other area (target), the source training data may not represent the target data. Two domain shifts are generally recognized.^25^ They are (1) covariate shifts (*P*(*X*_s_) ≠ *P*(*X*_t_)), the distribution of covariates differs in source and target data; and (2) conditional shifts (*P*(*Y*_s_|*X*_s_) ≠ *P*(*Y*_t_|*X*_t_)), the associations between predictor features and the response differs in source and target domains.

Coral is primarily designed to address covariate shifts without target labels while assuming no conditional shifts. Target labels stand for *Y*_t_ which is the air pollution measurements in the target area. To illustrate the concept of covariate shifts, we can consider a covariate feature–the annual average number of cars on a road segment (traffic intensity). For instance, if we assume that traffic intensities in a pseudo source area vary from 2000 to 5000 vehicles per day, but in a pseudo target area, the range extends from 5000 to 10,000. The model trained in the source area performs well within the range of 2000–5000 vehicles. However, it exceeds the range encountered during the training when forcing it to predict roads in the target area with 10,000 vehicles. Consequentially, the model can be inaccurate as models have a good generalization only if instances are seen during training. Coral transforms the covariates into a comparable distribution by encoding both the source and target features into a common space. Differently, conditional shifts occur when the relationships between predictor variables and the response in target and source areas vary. In other words, conditional shifts occur when the two areas’ overall emission patterns are different. For instance, in streets with equivalent traffic intensity, one area may predominantly consist of electric cars, while another is characterized by diesel trucks. In such a scenario, applying a model developed with electric cars to predict air pollution in the area with trucks would lead to an underestimation of air pollution levels.

#### 2.2.2 IDW_Coral

**Figure 1:**
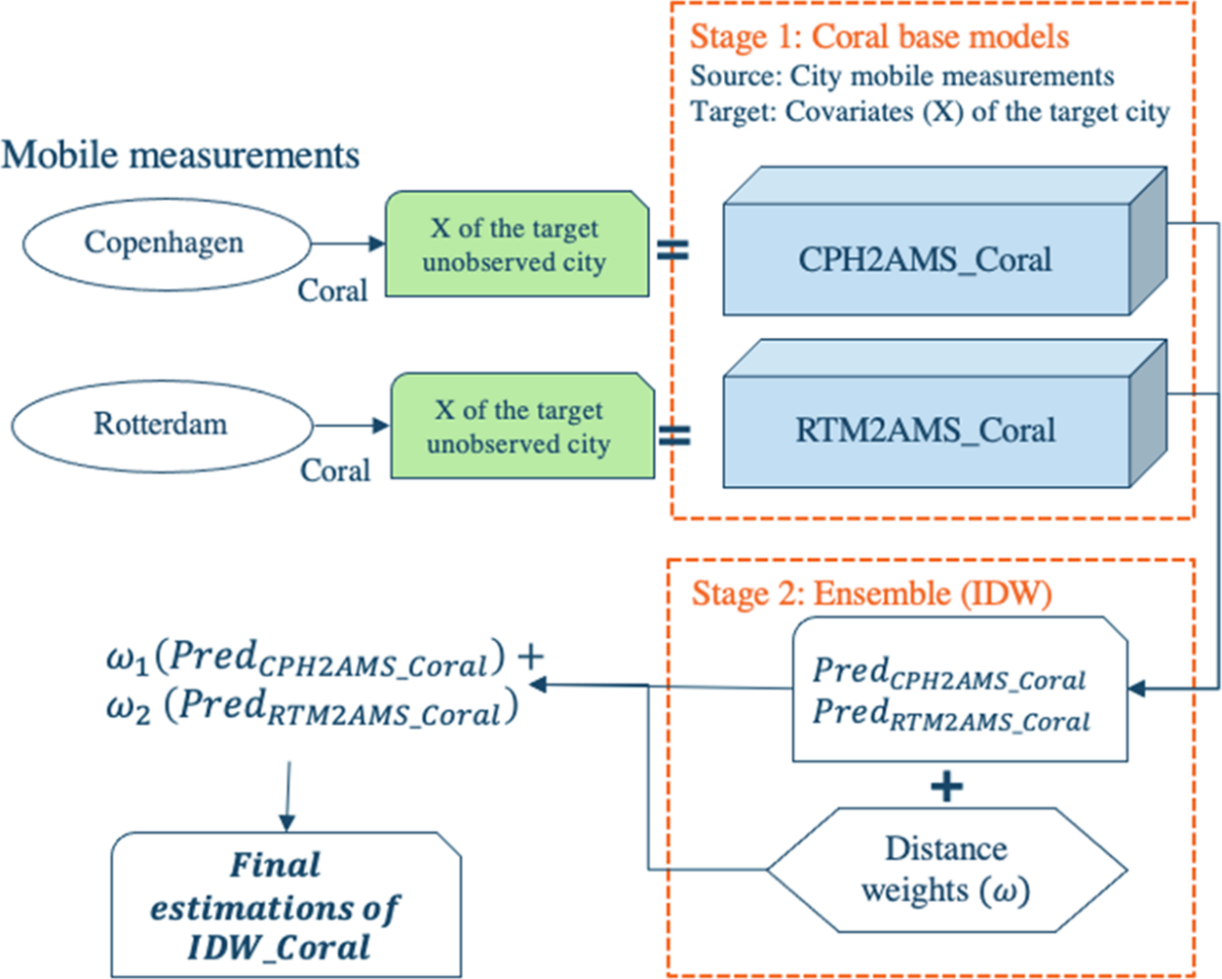
Modeling process diagram of IDW_Coral. CPH, RTM, and AMS are the abbreviations of Copenhagen, Rotterdam, and Amsterdam, respectively. CPH2AMS_Coral presents applying the transfer learning algorithm (Coral) to transfer knowledge from Copenhagen to estimate air pollution levels in Amsterdam. IDW_Coral weights the predictions of CPH2AMS_Coral and RTM2AMS_Coral by the inverse spatial distances.

Mitigating conditional shifts is challenging when target labels (measurements) are unavailable for statistical learning models. However, according to Tobler’s first law of geography, most natural objects and phenomena including the absolute levels and the emission pattern of air pollution are more likely to be similar to those of nearby areas.^26^ Therefore, to alleviate the impact of conditional shifts, an intuitive approach involves leveraging spatial distances to weight individual Coral models (IDW_Coral), like the classic spatial interpolation algorithm Inverse Distance Weighting (IDW). Instead of interpolating observation values, IDW_Coral interpolates individual Coral models. The single Coral models transferred from nearby monitoring areas are weighted higher and the far-away areas are weighted less. In the end, the information/knowledge from all mobile monitoring areas is summed. In this paper, IDW_Coral was implemented by integrating predictions from CPH2AMS_Coral and RTM2AMS_Coral weighted by their inverse spatial distances (conceptualized in Figure 1). Note that the weights are homogeneously applied to the prediction of CPH2AMS_Coral and RTM2AMS_Coral because the conditional shifts only differ between cities. The code of IDW_Coral is publicly available at https://github.com/ZhendongYuan/transfer_mapping.

### 2.3 Directly Applied LUR Models

Stepwise linear regression (SLR) LUR models are often used for air pollution mapping.^4,27,28^ SLR assumes a linear relationship between predictor features and air pollution measurements. It selects predictor features in a forward stepwise manner to avoid collinearity. Details of SLR implementation were provided in Appendix Text S1. SLR models trained in the source areas (e.g., Copenhagen and Rotterdam) and directly applied to the target area (i.e., Amsterdam) were labeled as the directly applied LUR models (Table 3). In this context, two SLR models were implemented, namely CPH2AMS_SLR and RTM2AMS_SLR. These models were trained using the predictor features from the source area and applied to predict air concentrations in Amsterdam using predictor features from Amsterdam. Additionally, we integrated these two directly applied SLR models (i.e., CPH2AMS_SLR and RTM2AMS_SLR) into the IDW framework, leading to the IDW_SLR model. Compared to IDW_Coral, IDW_SLR lacks the transfer learning component, serving as an indicator of the efficiency of the transfer learning algorithm.

**Table 3:** Summary of Models Implemented^a^^,^^b^

**model category**	**model name**	**model input**	**algorithms**
local LUR model	AMS_SLR	Amsterdam mobile measurements with sequentially increasing collection days (*X*_t_,*Y*_t_)	SLR
transfer learning LUR model	CPH2AMS_Coral	RTM/CPH 30-day mobile data (*X*_s_,*Y*_s_) + Predictor features of Amsterdam (*X*_t_)	Coral
	RTM2AMS_Coral		
	IDW_Coral	distance weighted predictions of (RTM2AMS_Coral + CPH2AMS_Coral)	inverse distance weighted Coral
directly applied LUR model	CPH2AMS_SLR	CPH 30-day mobile data only (*X*_s_,*Y*_s_)	directly applied SLR
	RTM2AMS_SLR	RTM 30-day mobile data only (*X*_s_,*Y*_s_)	
	IDW_SLR	distance weighted predictions of (RTM2AMS_ SLR + CPH2AMS_ SLR)	inverse distance weighted SLR

a *X*_s_ denotes the predictor variable such as land use, traffic and population in the source area. *X*_t_ represents the target area. *Y*_s_ denotes the air pollution in the source area. *Y*_t_ presents the air pollution levels in the target area. In the context of transfer learning, *Y*_t_ is also called the target labels.

b RTM: Rotterdam; CPH: Copenhagen; AMS: Amsterdam; CPH2AMS: models transfer mobile measurements in Copenhagen to estimate air pollution levels in Amsterdam. Same for RTM2AMS.

We chose SLR as the representation of the city-specific LUR model instead of machine learning algorithms such as random forest (RF), because we found previously that RF is generally less generalizable than SLR in our modeling of air pollution in Amsterdam and Copenhagen.^11,29^ More complex models need to tune more parameters during training which can be significantly affected by the domain differences.^11^

### 2.4 Local Reference Models

Mobile measurements in Amsterdam were collected for 160 days. These measurements were sliced into different collection days, sequentially increasing from 1 to 160 days. Each slice was resampled 20 times. A series of SLR models were fitted using each data slice (AMS_SLR, Table 3).

## 3 Results and Discussion

This study analyzed mobile measurements collected from three different European cities. There is no spatial overlap between the three cities. Rotterdam is located 57km (straight-line distance) from Amsterdam, while Copenhagen is 620km from Amsterdam. We demonstrated that by transferring mobile measurements from Copenhagen and Rotterdam, IDW_Coral showed the capability to estimate accurate air pollution in Amsterdam while preserving the hyperlocal spatial variations. Without involving any local measurements, IDW_Coral achieved MAE and RMSE comparable to those of local LUR models trained using the local mobile measurements. IDW_Coral substantially outperformed the directly applied LUR models developed in Rotterdam and Copenhagen without transfer learning algorithms. Additionally, IDW_Coral correlated strongly with our previously published mixed-effect models fitted using all Amsterdam mobile measurements.

### 3.1 External Validation for NO_2_

Integrating CPH2AMS_Coral and RTM2AMS_Coral models, IDW_Coral showed the most balanced performance between *R*^2^ and absolute errors, based on independent long-term monitoring measurements (Table 4). These measurements represent long-term (annual) air pollution concentrations more than cross-validation results based on short-term or on-road mobile measurements.^11,12^ Note that most other mobile monitoring campaigns are unable to perform such analysis due to the lack of external long-term validation data.

**Table 4:** Model Accuracy Validated by the Fixed-site Routine Monitors for NO_2_ (*n* = 82)

		external long-term validation		
model category	model name	*R*^2^	MAE (μg/m^3^)	RMSE (μg/m^3^)
feature-based transfer learning LUR models	CPH2AMS_Coral	0.39	6.47	7.61
	RTM2AMS_Coral	0.30	4.57	5.47
	IDW_Coral	0.35	4.47	5.36
directly applied SLR	CPH2AMS_SLR	0.17	7.86	10.77
	RTM2AMS_SLR	0.19	5.19	6.67
	IDW_SLR	0.21	4.94	6.28
local reference model	AMS_SLR_160D^a^	0.52	3.72	4.70
	AMS_SLR_30D^a^	0.39	4.83	5.47

a AMS_SLR_160D is the SLR model trained using Amsterdam mobile measurements collected for 160 days. AMS_SLR_30D is trained with 30 days of mobile measurements.

*R*^2^ reflects the goodness-of-fit (i.e., how much variance can be explained). Performance estimation needs to consider also the absolute errors. Notably, IDW_Coral achieved MAE and RMSE comparable to a locally fitted LUR model (AMS_SLR) developed using Amsterdam mobile measurements collected for 160 days. Specifically, it achieved an MAE and RMSE of 4.47 and 5.36 μg/m^3^ for NO_2_, which is 16 and 19% of the mean long-term average measurements (Palmes). Compared with the local LUR model trained with NO_2_ measurements of 160 collection days, differences were quite small (for MAE, IDW_Coral - AMS_SLR_160D = 0.75 μg/m^3^; for RMSE, IDW_Coral - AMS_SLR_160D = 0.66 μg/m^3^). IDW_Coral is as accurate as the local LUR model developed using local mobile measurements in terms of absolute errors. The *R*^2^ of IDW_Coral is mainly limited by the small variation of its predictions which are compressed between 25 and 35 μg/m^3^. While long-term average measurements of NO_2_ (Palmes) vary from 15 to 45 μg/m^3^ (Appendix Figure S1).

Previous studies rarely focus on the transferability of air pollution measurements. We found no literature targeting the application of a Land Use Regression (LUR) model based on mobile-monitored NO_2_ data from another city. Limited attempts exist for transferring fixed-site NO_2_ measurements. For instance, Poplawski et al.,^7^ transferred passive sampler measurements from Vancouver (Canada, 2 weeks, *n* = 116) to Victoria (Canada) and Seattle, using 50 and 26 local measurements in Victoria and Seattle for local calibrations. Their locally calibrated models achieved an *R*^2^ of 0.58 in Victoria and 0.65 in Seattle. Although the *R*^2^ values of our proposed mobile measurements-based models are not as high as those of fixed-site measurements-based models, mobile measurements can capture finer-scale spatial variations which might be more relevant to the application of human exposure assessment.

The performance of IDW_Coral (*R*^2^ = 0.35) was substantially better than the directly applied models developed in Rotterdam and Copenhagen without transfer learning algorithms, specifically CPH2AMS_SLR (*R*^2^ = 0.17), RTM2AMS_SLR (*R*^2^ = 0.19) and IDW_SLR (*R*^2^ = 0.21). The transfer learning algorithms and the IDW strategy contributed to its performance, as individual Coral models outperformed the directly applied LUR models and IDW_Coral was better than Coral models based on a single city.

**Figure 2:**
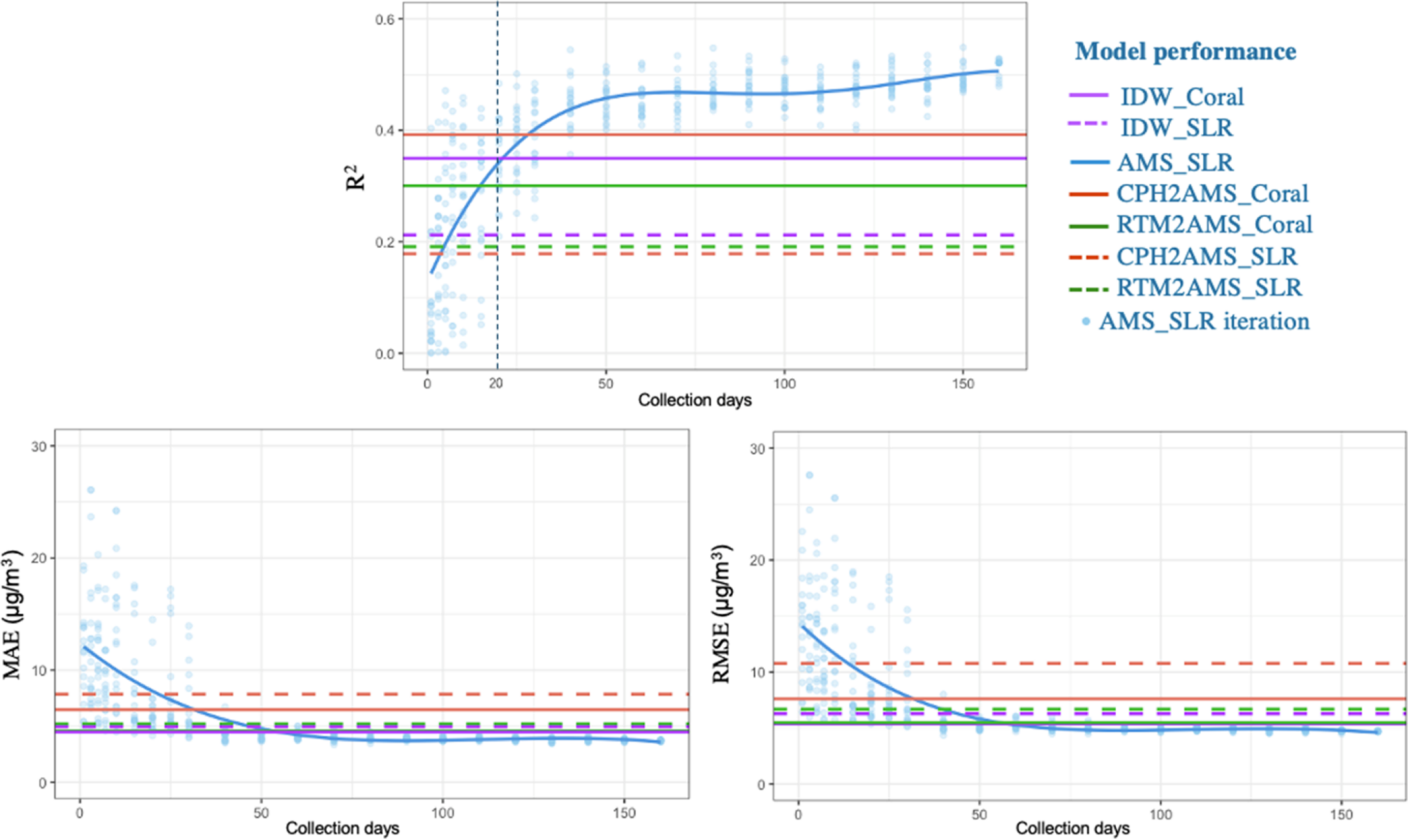
Comparison of overall model performance of NO_2_ predictions validated by the 82 fixed-site routine measurements in Amsterdam. AMS_SLR is the reference model fitted with gradually increased Amsterdam local mobile measurements. Each time slice was resampled 20 times and plotted as dots. The other models rely on mobile measurements only from existing mobile campaigns (30 days) conducted in Copenhagen and Rotterdam to estimate air pollution levels in Amsterdam.

The *R*^2^ of IDW_Coral achieved 67% of AMS_SLR_160D (*R*^2^ = 0.35 versus 0.52) and 90% of AMS_SLR_30D (*R*^2^ = 0.35 versus 0.39). AMS_SLR_160D refers to the local reference model trained using all mobile measurements in Amsterdam, while AMS_SLR_30D was trained using measurements collected within 30 days, which corresponds to the number of days available for Copenhagen and Rotterdam in this application. IDW_Coral without target measurements achieved an *R*^2^ similar to AMS_SLR when using data from 20 collection days (Figure 2). Meanwhile, for the MAE and RMSE, IDW_Coral equals AMS_SLR using data of 50 collection days. This implies that at least in Amsterdam, mobile campaigns of less than 20 days did not result in a better model than transferring knowledge from other areas using IDW_Coral. Consequently, to more accurately estimate long-term air pollution concentrations, mobile monitoring campaigns in Amsterdam need to span more than 20 days to gather city-specific insights. IDW_Coral presents a time and cost-effective alternative if this condition is not met.

#### 3.1.1 Transfer Learning Aspect

As introduced in Section 2, when training and applying LUR models in different areas, the probability distribution of covariates and the association between covariates and the response can differ. This reflects two domain shifts (covariate shifts and conditional shifts). They jointly affect the accuracy of the directly applied SLR models (i.e., CPH2AMS_SLR and RTM2AMS_SLR).

Coral models can partially reduce the covariate shifts. CPH2AMS_Coral and RTM2AMS_Coral improved *R*^2^ significantly, compared to the directly applied SLR model (Figure 2). Aligning the source and target feature space makes the training cover the “unseen” situations in the target data, effectively reducing extreme values in predictions. This, in turn, reduced the variability of residuals for both CPH2AMS_Coral and RTM2AMS_Coral, as indicated in the scatter plot in Appendix Figure S1. Therefore, compared to the directly applied LUR models, not only has the *R*^2^ improved significantly, but there is also a decrease in absolute errors, particularly for CPH2AMS_Coral.

The Coral method cannot correct the conditional shifts without target labels. Conditional shifts occur, when the emission pattern exhibits significant distinctions between source and target areas which means the associations between covariates and the response can be different (*P*(*Y*_s_*|X*_s_) *≠ P*(*Y*_t_|*X*_t_)). If we decompose the conditional distribution *P*(*Y*|*X*) into *P*_general_(*Y*|*X*) and *P*_cityspecific_(*Y*|*X*), we can assume *P*_general_(*Y*|*X*) universal across different areas and *P*_cityspecific_(*Y*|*X*) is varying across areas. This was clear when comparing the coefficients between SLR models trained in three cities (Appendix Tables S3–S5). Covariates related to traffic, population, and port were commonly selected. The similar coefficient structure reflects a similar *P*_general_(*Y*|*X*). However, different magnitudes of coefficients applied to different cities, suggesting distinct *P*_cityspecific_(*Y*|*X*). Ignoring the conditional difference of *P*_cityspecific_(*Y*|*X*), the Coral models may experience higher levels of uncertainty, particularly concerning absolute errors. For example, the CPH2AMS_Coral yielded a notable MAE of 6.47 μg/m^3^.

**Figure 3:**
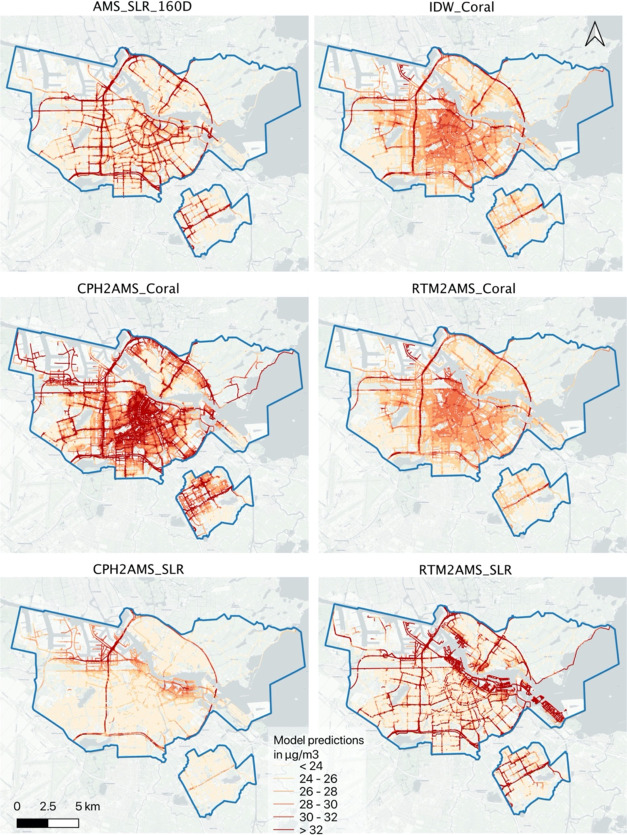
Spatial maps of NO_2_ predictions. Their differences compared to the local reference model - AMS_SLR_160D are plotted in the appendix Figure S2.

Rotterdam is only 57 km from Amsterdam. By geographical laws, closer locations tend to exhibit greater similarity both in the built-up area and airshed. Despite some localized differences, the emission pattern of air pollution in Rotterdam could be generally similar to those in Amsterdam. It indicates that its conditional distribution may also be partially similar to the situation in Amsterdam. Even without transfer learning methods, directly applying the LUR model - RTM2AMS_SLR achieved low absolute errors (low MAE and RMSE in Figure 3) but a low *R*^2^ due to the covariate shifts. Targeting covariate shifts, the RTM2AMS_Coral exhibited significantly improved *R*^2^ compared to the RTM2AMS_SLR but only marginally in MAE and RMSE.

#### 3.1.2 Inverse Distance Weighted Assemble Strategy

The framework of IDW can mitigate the drawbacks of the individual Coral models mentioned above. Through reweighting based on distances, IDW_Coral assigns a high degree of importance, approximately 9/10, to the knowledge derived from Rotterdam compared to Copenhagen. Since the conditional distribution *P*_cityspecific_(*Y*|*X*) in Rotterdam is more similar to Amsterdam than Copenhagen, conditional shifts in IDW_Coral are partially reduced. Meanwhile, the general knowledge component *P*_general_(*Y*|*X*) is further enhanced in IDW_Coral as more mobile measurements from another area are incorporated, increasing the diversity (fewer “unseen” instances). Therefore, the IDW_Coral obtained a lower MAE and RMSE than single Coral models. Additionally, averaging knowledge in Copenhagen and Rotterdam resulted in an intermediate *R*^2^ between CPH2AMS_Coral and RTM2AMS_Coral.

Without Coral, IDW_SLR incorporates predictions from CPH2AMS_SLR and RTM2AMS_SLR models. It outperformed the individual CPH2AMS_SLR and RTM2AMS_SLR models (Table 4). However, IDW_SLR did not perform as well as IDW_Coral. This discrepancy can be attributed to IDW_Coral’s capacity to mitigate covariate shifts by integrating two transfer learning models.

#### 3.1.3 Spatial Pattern of NO_2_ Predictions

The hyperlocal intracity variations were preserved by models trained using mobile measurements. NO_2_ concentrations estimated by CPH2AMS_SLR and RTM2AMS_SLR show high concentrations mainly in the port/water area and along the major ring roads in Amsterdam which corresponded to their coefficients where port, water, and major-road-related features were among the most influential features (Appendix Tables S3–S5). NO_2_ concentrations estimated by the three Coral models preserved the major road’s high values and showed elevated NO_2_ concentrations in the city center. However, AMS_SLR_160D, trained with local measurements, presented a distinct spatial pattern. It indicated higher concentrations along major roads, without a discernible hotspot pattern in the city center.

Apart from the port and water areas, NO_2_ concentrations estimated by RTM2AMS_SLR visually resemble the distribution of AMS_SLR_160D in the remaining areas. This suggests partially similar emission patterns between Rotterdam and Amsterdam. However, significant overestimations by RTM2AMS_SLR in the port area emphasize variations in the city-specific patterns in this zone.

### 3.2 NO_2_ and UFP Predictions Compared with Mixed-effect Model

**Figure 4:**
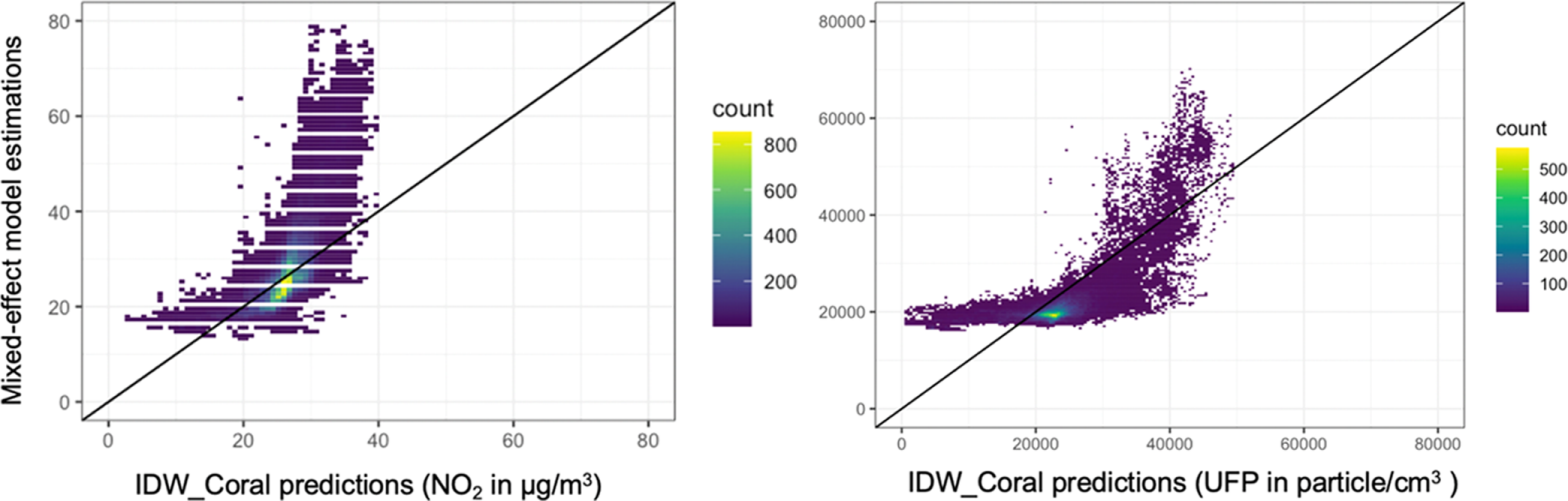
Scatter plot of IDW_Coral predictions for all road segments against estimations from mixed-effect models using Amsterdam mobile measurements of all collection days for NO_2_ (left) and UFP (right).

Model comparisons for NO_2_ and UFP between the tested models and the previously published mixed-effects models are summarized in Table 5. For NO_2_, AMS_SLR_160D achieved the highest Pearson correlation of 0.92 and CCC of 0.88 to the mixed-effect model trained using 160 collection days in Amsterdam. Meanwhile, IDW_Coral achieved a Pearson correlation of 0.72 and low MAE and RMSE. IDW_Coral tends to underestimate the predictions of the mixed-effect model, particularly at locations where the mixed-effect model estimated high concentrations (ranging from 20 to 40 μg/m^3^ in Figure 4). This can be attributed to a limitation inherent in Coral. As an unsupervised transfer learning algorithm, Coral aligns the feature space of the source and target, which unavoidably results in a compromise in the representation of extreme values. Interestingly, it appears that this limitation seems to compensate for the prediction inaccuracy of our mixed-effect models. In previous studies, we observed that mixed-effect models often overestimate true NO_2_ concentrations by approximately 25%, as mobile measurements are collected on-road as opposed to roadside routine validation measurements.^1^

**Table 5:** Model Comparisons with Previously Published Mixed-effect Model Estimates on All Road Segments in Amsterdam

pollutants	model categories	models	Pearson correlation^a^	CCC^b^	MAE	RMSE
NO_2_ μg/m^3^	local reference model	AMS_SLR_160D	0.92	0.88	3.18	4.44
	directly applied SLR	CPH2AMS_SLR	0.82	0.71	6.82	10.10
		RTM2AMS_SLR	0.77	0.38	6.03	9.02
		IDW_SLR	0.82	0.50	5.58	8.14
	feature-based TL LUR models	CPH2AMS_Coral	0.76	0.75	5.15	6.82
		RTM2AMS_Coral	0.67	0.43	4.89	7.91
		IDW_Coral	0.72	0.50	4.64	7.42
UFP particles/cm^3^	local reference model	AMS_SLR_160D	0.90	0.80	3736	4536
	directly applied SLR	CPH2AMS_SLR	0.89	0.46	13,076	18,168
		RTM2AMS_SLR	0.74	0.66	5149	7250
		IDW_SLR	0.79	0.70	5255	7180
	feature-based TL LUR models	CPH2AMS_Coral	0.60	0.13	10,357	11,727
		RTM2AMS_Coral	0.70	0.65	5089	6190
		IDW_Coral	0.71	0.69	4239	5461

a Pearson correlation primarily measures the strength and direction of a linear relationship between two variables. All P-values were less than 2.2 × 10^–16^.

b CCC - concordance correlation coefficient. It reflects the overall agreement between two sets of variables without a linear assumption.

Consistent with the external validation results for NO_2_ (Section 3.1), despite Coral models exhibiting a lower Pearson correlation than the directly applied LUR models, the higher CCC values in Table 5 indicate higher levels of agreement (without linear assumptions) to the mixed-effect model predictions. The proposed Coral models consistently yield lower MAE and RMSE than directly applied LUR models. This confirms the effectiveness of transfer learning to improve the prediction capability, even without local measurement, which is considered the most crucial feature for large-scale air pollution mapping.

Due to the absence of long-term validation data sets for UFP in Amsterdam, IDW_Coral predictions were only compared to the UFP estimations from the mixed-effect model. A Pearson correlation of 0.71 was observed between these two models, similar to NO_2_ (*r* = 0.72). Although the mixed effect model does not serve as a ground truth, the strong correlation indicates a similarity in the concentration patterns predicted by both models. IDW_SLR slightly correlates better with the mixed-effect model predictions than IDW_Coral for UFP and IDW_SLR achieved smaller absolute errors (lower MAE and RMSE). Their marginal differences in correlations and absolute errors (Table 5) make it difficult to differentiate IDW_SLR from IDW_Coral. We found a lower correlation of the CPH2AMS_Coral model (compared to the directly applied CPH2AMS_SLR model), leading to a relatively lower correlation of the IDW_Coral as well. However, we attribute this to the fact that the correlation of the directly applied SLR model is artificially high. Specifically, the CPH2AMS_SLR predicted concentrations up to 191,372 particles/cm^3^, creating a very large variance. This variance was significantly higher than the mixed-effect model’s predictions, where a maximum of 70,200 particles/cm^3^ was predicted. Because the Coral model mimics the distribution of the target features, the CPH2AMS_Coral predicted the lowest maximum concentration (36,805 particles/cm^3^; Appendix Table S2). In other words, the higher Pearson correlation of CPH2AMS_SLR is mainly caused by its significantly higher prediction variations than those of the mixed-effect models. Nevertheless, the overall accuracy, measured by MAE and RMSE, indicates that CPH2AMS_Coral outperforms CPH2AMS_SLR.

In the literature, locally calibrated linear-regression-based LUR models are the most recommended methods to transfer measurements between cities. They are designed to retain the model structure developed in one area, while only recalibrating the coefficients based on some measurements in the target area. Patton et al.,^9^ implemented locally calibrated transferred models to transfer mobile monitored particle number concentration among urban neighborhoods in the Boston area, achieving *R*^2^ values of 0.19–0.40. Zalzal et al.,^8^ applied a similar locally calibrated transferred model to transfer UFP mobile measurements between Toronto and Montreal, achieving *R*^2^ values of 0.36 for Toronto and 0.38 for Montreal. Note that both validations are based on mobile measurements, while our validation based on the predictions of the mixed-effect models better represents the true distribution of concentrations than purely mobile measurements. Our proposed IDW_Coral model achieved a Pearson correlation of 0.71 with the 50m road segment UFP concentration estimates of the mixed-effect model which equals an *R*^2^ of 0.5 which is higher than the accuracy reported in the other studies in terms of UFP. Note, IDW_Coral is developed without utilizing any measurement (mobile or fixed-site) in Amsterdam.

**Figure 5:**
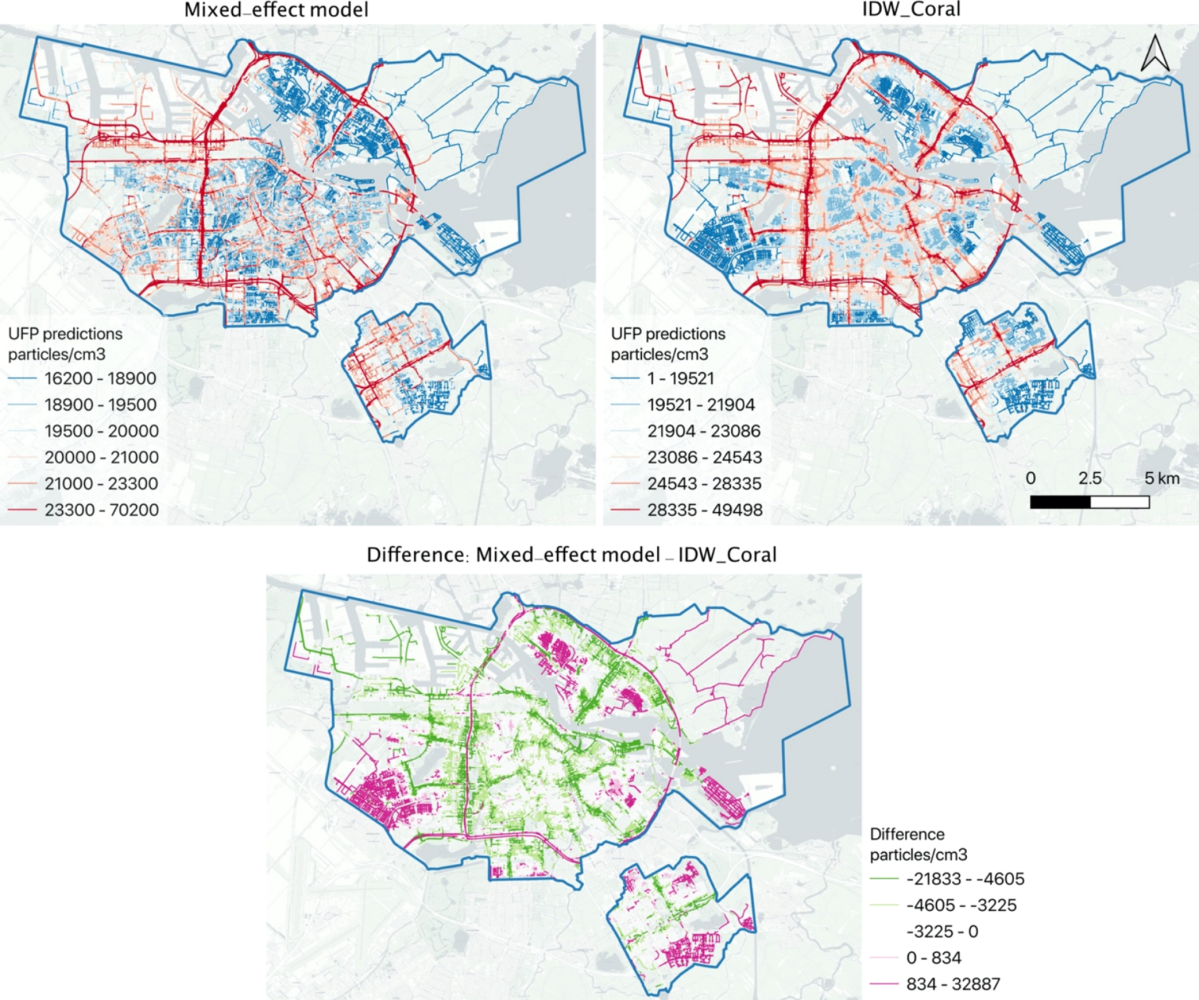
Top left: Spatial map of UFP estimations from the mixed-effect model (publicly available in Google Insights Explorer^21^). Top right: The predictions from IDW_Coral. Bottom: The difference map (*Mixed*_*effect*-*IDW*_*Coral*). Spatial maps of UFP predictions with a unified legend are plotted in Appendix Figure S3.

The spatial maps of the mixed-effect and IDW_Coral models were colorized by the equal-quantile method to better illustrate their intracity variations (Figure 5). Despite the different levels of absolute values, the general spatial pattern of IDW_Coral UFP predictions was similar to the mixed-effect model predictions, with the distinction that IDW_Coral predicts slightly higher concentrations in the city center and lower concentrations on the major ring roads and suburbia residential areas (shown in the difference map in Figure 5).

### 3.3 Strength and Limitation

The following characteristics of the proposed IDW_Coral approach were identified.

We illustrated the feasibility of transferring the mobile knowledge from existing mobile campaigns to map hyperlocal air pollution for a city without any air pollution measurements. Compared to the other transferred models in the literature where recalibration is performed,^8,9^ IDW_Coral requires no measurements in the target area. IDW_Coral shows improvement over the directly applied LUR models, particularly for NO_2_. Compared to the local NO_2_ reference model, IDW_Coral achieved 67% of the performance (based on *R*^2^) compared to the AMS_SLR with 160 collection days and comparable MAE and RMSE. The hyperlocal variations of air pollution are preserved by IDW_Coral incorporating fine-grained mobile measurements from Copenhagen and Rotterdam. This is mainly attributed to the design of the end-to-end Coral algorithm, which directly bridges covariates between the target and source domains and reduces the impacts of differences in covariates across space and time.

Scalability. This work used two existing mobile monitoring cities as the source for transfer learning. Like spatial interpolation, expanding the coverage of mobile-monitored areas, the IDW_Coral will be more accurate with the potential for broader applicability as more diverse situations will be involved. With only a few European cities currently mobile monitored, our proposed IDW_Coral model can produce an accurate air pollution map in fine spatial resolution across Europe which can significantly save the efforts of conducting mobile monitoring campaigns throughout numerous European cities.

Flexibility. The proposed framework can be flexibly adapted by different transfer learning algorithms. For example, we used Coral as one of the most commonly applied unsupervised transfer learning approaches. However, other unsupervised transfer learning algorithms exist, such as kernel-based method - Kernel Mean Matching (KMM) and neural-network-based – Discriminative Adversarial Neural Network (DANN). These methods could be compared to Coral in future work. We demonstrated the enhanced model performance of distance-inverse weighting strategies. As the first study of its kind, our work demonstrates a potential feasible direction in this field. Exploring various weights, such as dispersion applicable indices or even learned weights, could enhance the depiction of similarities, potentially leading to further improvement of the mapping accuracy. Such weighting mechanisms may have the advantage of embedding the physiochemical characteristics and reflecting more regional information that cannot easily be operationalized as predictor features, such as policy differences, local climate zones, chemical transport models, or even fixed-site monitoring measurements.

The following limitations of this study are acknowledged. First, mobile measurements from only two monitored cities were used in the IDW_Coral, resulting in only a moderate *R*^2^ value in the external long-term validation. Whether an *R*^2^ value is adequate depends on the model’s application and the available alternatives. For the current scarcity of UFP measurements, imperfect models provide a foundation for societal discussions and further research. This accuracy can be further improved with more diverse measurements collected from more areas. However, validating this conjecture is challenging as it requires conducting more mobile campaigns to cover larger areas which is time-consuming and costly. Second, a common drawback of Coral is that the predictor variables in both the source and target cities must be the same. When more cities have been monitored and included in the framework, harmonizing the predictor variables demands extra effort. Third, although Coral helps to bridge the domain difference between the source and target areas, the knowledge transferred is still learned from the on-road short-term mobile measurements. Its representativeness of the target long-term concentrations at residential locations might be biased as we have demonstrated previously.^11,12^ Fourth, our predictor variables do not include meteorological information. However, this information is rarely available in fine spatial resolution (i.e., 50m road segments) and their variations are too small to be meaningful as opposed to the larger scale. Future work can investigate whether adding coarse meteorology information (such as the finest EURO1K^30^ in 1KM*1KM) can benefit our models. Fifth, we acknowledge that cities differ markedly in multiple domains, including emission sources, urban configuration, and climate, limiting direct transferability of models from one city to another city. Although our IDW-CORAL approach builds on this limitation, the uncertainties raised from these differences are only partially reduced. Sixth, UFP predictions lack validation using external routine monitors. The predictions produced by the Mixed-effect model are not the ground truth. Future work should conduct long-term fixed-site UFP monitoring campaigns in Amsterdam to produce a more reliable benchmark.

Our study highlights the benefit of leveraging geographic principles in transferring and fusing knowledge from diverse mobile monitoring campaigns to map local air pollution. IDW_Coral outperformed the direct application of Copenhagen and Rotterdam LUR models and achieved MAE and RMSE comparable to a locally fitted LUR model (AMS_SLR), developed using Amsterdam mobile monitoring data from 160 collection days for NO_2_. The *R*^2^ of IDW_Coral was similar to that of the AMS_SLR based on 20 collection days, and the MAE and RMSE of IDW_Coral equals AMS_SLR using data of 50 collection days. This suggests that a mobile campaign in Amsterdam lasting less than 20 days may be of limited value, as IDW_Coral can serve as an equally accurate alternative. IDW_Coral exhibited a relatively high Pearson correlation (0.72 for NO_2_ and 0.71 for UFP) with mixed-effect models incorporating all Amsterdam local mobile measurements. Similar to the classic spatial interpolation algorithm (i.e., IDW), the performance and scalability of IDW_Coral can be further enhanced by expanding mobile monitored areas and further developing weights beyond a simple distance-based similarity measure. IDW_Coral demands no direct measurements in the target area, showcasing its potential for large-scale applications in European urban areas, resulting in significant economic efficiencies in executing mobile monitoring campaigns throughout numerous European cities.

## Supporting Information Available

The appendix includes text describing the developing steps of the SLR model, tables summarizing the predictor variables, UFP model predictions, and the SLR models trained in three European cities, as well as figures of a scatterplot of IDW_Coral predictions against Palmes validation measurements, NO_2_ maps showing differences among model predictions, and UFP maps comparing mixed-effect and IDW-Coral model predictions (PDF)
