# Supplementary material for: Hyperlocal Air Pollution Mapping: A Scalable Transfer Learning LUR Approach for Mobile Monitoring
Source: Environ Sci Technol. 2024 Jul 31;58(32):14372–83. doi: 10.1021/acs.est.4c06144 (PMC11325550; doi:10.1021/acs.est.4c06144)
Supplement: Supplementary file 1 — es4c06144_si_001.pdf [file es4c06144_si_001.pdf]

Supplementary Information for:

Hyperlocal Air Pollution Mapping: A Scalable Transfer Learning LUR Approach for Mobile Monitoring

Zhendong Yuan[1], Jules Kerckhoffs [1], Hao Li [2], Jibran Khan[3,4], Gerard Hoek [1], Roel Vermeulen[1,5]*

[1]Institute for Risk Assessment Sciences, Utrecht University, 3584 CM Utrecht, Netherlands

[2] Professorship of Big Geospatial Data Management, Technical University of Munich, 85521 Ottobrunn, Germany

[3]Department of Environmental Science, Aarhus University, DK-4000 Roskilde, Denmark

[4]Danish Big Data Centre for Environment and Health (BERTHA), Aarhus University, DK-4000 Roskilde, Denmark

[5]Julius Centre for Health Sciences and Primary Care, University Medical Centre, Utrecht University, 3584 CX Utrecht, The Netherlands

*Corresponding author

z.yuan@uu.nl

This file contains one text, five tables and three figures.

Text:

Text S1. Model implementation of SLR

Tables:

Table S1. Spatial predictor variables with units, predefined directions of effect, and buffer sizes in AMS.

Table S2. Statistics of UFP model predictions in particle/cm^3.

Table S3. SLR trained in Rotterdam.

Table S4. SLR trained in Copenhagen.

Table S5. SLR trained in Amsterdam.

Figures:

Figure S1. Scatterplot of model predictions against fixed-site validation measurements.

Figure S2. NO_2 maps of differences between SLR_AMS_160D and the other model tested.

Figure S3. Spatial maps of UFP predictions from the mixed-effect and IDW_Coral models with a unified legend.

Text S1. Model implementation of SLR

The stepwise linear regression (SLR) model selects predictor variables in a forward stepwise manner. It starts by taking an empty, intercept-only model, and then adds variables based on the goodness of fit determined via the adjusted R^2 values. The variable having the highest adjusted R^2 value was added first in the model, and the model development process stopped when adding new variables could not improve the adjusted R^2. Predictor variables are only included when the direction of the association is predetermined (e.g., positive for traffic load). The predictor variables in the LUR models were checked for p-value and collinearity. Here, p-value > 0.10 and variance inflation factor > 3 and Cook's D <1 was removed.

Table S1: Spatial predictor variables with units, predefined directions of effect, and buffer sizes in AMS.

Predictor variable	Abbreviation	Units	Direction of effect	Buffer	10th percentile	Mean	90th Percentile
**Agricultural land area**[1]	AGRI_	m^2	-	**100**	0	1159	0
				**300**	0	11288	0
				**500**	0	34779	64052
				**1000**	0	178942	649727
				**5000**	1710991	13290916	30287613
**Airport area**[1]	AIR_	m^2	+	**5000**	0	640794	1628347
**Industry area**[1]	INDUS_	m^2	+	**100**	0	2006	0
				**300**	0	17769	41347
				**500**	0	49074	180131
				**1000**	0	199831	730248
				**5000**	2902185	5182292	8091464
**Natural and forested areas**[1]	NATUR_	m^2	-	**100**	0	0	0
				**300**	0	0	0
				**500**	0	0	0
				**1000**	0	0	0
				**5000**	0	429332	2388024
**Port area**[1]	PORT_	m^2	+	**100**	0	2648	2516
				**300**	0	22447	85979
				**500**	0	59770	258949
				**1000**	0	226393	956817
				**5000**	0	6167718	13267782
**Residential land area**[1]	RES_	m^2	+	**100**	0	21152	31416
				**300**	0	183950	282742
				**500**	0	492795	785396
				**1000**	104805	1826998	3040804
				**5000**	17043122	32157817	46811645
**Transportation area**[1]	TRANS_	m^2	+	**100**	0	0	0
				**300**	0	0	0
				**500**	0	13457	2874
				**1000**	0	50061	233924
				**5000**	519010	1340506	2093995
**Urban Green area**[1]	URBG_	m^2	-	**100**	0	2351	7952
				**300**	0	25250	102385
				**500**	0	79234	268822
				**1000**	0	361378	852803
				**5000**	6248972	9362179	13883066
**Water**[1]	WATER_	m^2		**100**	0	0	0
				**300**	0	10238	30716
				**500**	0	37897	152537
				**1000**	0	222478	741893
				**5000**	2904140	8362718	12310634

**Population density**[2]	POP_	n	+	**100**	0	319	770
				**300**	0	2385	5355
				**500**	25	6091	13410
				**1000**	1485	21762	46115
				**5000**	146514	366674	580006
**Traffic intensity on nearest road**[3]	TRAFNEAR	Veh/day	+		137	9986	22487
**Traffic intensity on nearest major road**[3]	TRAFMAJOR	Veh/day	+		3115	14212	28042
**Heavy-duty traffic intensity on nearest road**[3]	HTRAFNEAR	Veh/day	+		0	619	1333
**Heavy-duty traffic intensity on nearest major road**[3]	HTRAFMAJOR	Veh/day	+		54	980	2138
**Road length of all roads**[3]	RDL_	m	+	**25**	39	66	100
				**50**	100	187	294
				**100**	254	636	966
				**300**	2029	4880	7083
				**500**	7893	13761	19090
				**1000**	29092	50344	68095
**Road length of all major roads**[3]	MRDL_	m	+	**25**	0	24	91
				**50**	0	69	198
				**100**	0	210	534
				**300**	0	1532	3554
				**500**	0	3787	8800
				**1000**	3015	13627	31882
**Traffic intensity on all roads (sum of (traffic intensity * length of all segments))** [3]	TLOA_	(Veh/day)*m	+	**25**	0	634722	1579945
				**50**	6393	1815133	4797110
				**100**	78159	5494027	14290643
				**300**	3038453	36918277	1.01E+08
				**500**	13741938	44180744	87625101
				**1000**	62971674	166892417	324383228
**Traffic intensity on all major roads (sum of (traffic intensity* length of all segments))** [3]	TMLOA_	(Veh/day)*m	+	**25**	0	543194	1387293
				**50**	0	1548534	4473085
				**100**	0	4550786	13415227
				**300**	0	12783795	26453420
				**500**	0	30877644	64537936
				**1000**	26948221	116664854	238567679
**Heavy-duty traffic intensity on all roads (sum of (heavy-duty traffic intensity* length of all segments))** [3]	HLOA_	(Veh/day)*m	+	**25**	0	40889	59383
				**50**	0	115926	205867
				**100**	1607	336918	909792
				**300**	84212	2085219	8507687
				**500**	387896	2664654	6027990
				**1000**	2550331	10515869	25699398
**Heavy-duty traffic intensity on major roads (sum of (heavy-duty traffic intensity*length of all segments)** [3]	HMLOA_	(Veh/day)*m	+	**25**	0	35363	41295
				**50**	0	100488	141074
				**100**	0	286355	742416
				**300**	0	846066	1614863
				**500**	0	2033997	4769469
				**1000**	966747	8018542	22978460

[1]*Source: CORINE (Copernicus Land Monitoring Service) 2018, raster 100m.*

[2]*Source: CBS (Central Bureau of Statistics Netherlands) 2017, 100m*100m.*

[3]*Source: NWB (National Road Network Netherlands) 2011, vehicles per day per segment (between intersections)*

Table S2: Statistics of UFP model predictions in particle/cm^3.

**Model names**	***Min.***	***1st Qu.***	***Median***	***Mean***	***3rd Qu.***	***Max***
Mixed-effect model	16,200	19,200	20,000	22,175	21,800	70,200
AMS_SLR_160D	15,444	21,722	23,587	25,588	26,810	70,903
CPH2AMS_SLR	-1,543	5,723	9,796	16,941	17,556	191,372
RTM2AMS_SLR	7,123	16,164	19,701	23,151	26,785	79,853
IDW_SLR	7,642	15,353	18,886	22,629	26,161	88,468
CPH2AMS_Coral	5,249	10,410	11,100	11,832	12,397	36,805
RTM2AMS_Coral	2.42	22,043	24,218	24,865	27,394	52,094
IDW_Coral	1.01	21,102	23,102	23,762	26,014	49,497

Table S3: SLR trained in Rotterdam

	Estimate	***Std. Error***	***t value***	***Pr(>|t|)***	
**(Intercept)**	7.598e+00	1.297e-01	58.558	<2e-16	***
**PORT_5000**	6.990e-08	8.808e-09	7.936	2.13e-15	***
**PORT_1000**	1.603e-06	9.488e-08	16.890	<2e-16	***
**MRDL_100**	1.879e-03	1.149e-04	16.348	<2e-16	***
**WATER_5000**	7.356e-08	4.736e-09	15.532	<2e-16	***
**RES_5000**	4.877e-08	3.356e-09	14.532	<2e-16	***
**INDUS_5000**	1.398e-07	1.567e-08	8.917	<2e-16	***
**NATUR_5000**	3.064e-08	1.551e-08	1.975	0.0483	*
**TRANS_1000**	1.419e-06	1.864e-07	7.612	2.74e-14	***
**AGRI_100**	3.803e-05	3.039e-06	12.515	<2e-16	***
**INDUS_500**	1.289e-06	2.381e-07	5.412	6.25e-08	***
**WATER_500**	1.574e-06	3.617e-07	4.353	1.34e-05	***
**URBG_100**	5.097e-06	4.652e-06	1.096	0.2732	
**TRAFNEAR**	4.782e-05	1.716e-06	27.866	<2e-16	***
**Multiple R-squared: 0.08009, Adjusted R-squared: 0.07988**					

Signif. codes: 0 '***' 0.001 '**' 0.01 '*' 0.05 '.' 0.1 ' ' 1

Table S4: SLR trained in Copenhagen

	Estimate	***Std. Error***	***t value***	***Pr(>|t|)***	
**(Intercept)**	-1.844e-01	5.297e-01	-0.348	0.727717	
**TRANS_5000**	1.994e-06	1.158e-07	17.223	<2e-16	***
**PORT_300**	2.572e-05	1.483e-06	17.343	<2e-16	***
**WATER_1000**	7.235e-06	4.429e-07	16.335	<2e-16	***
**POP_100**	3.360e-03	5.600e-04	5.999	2.03e-09	***
**TRANS_1000**	4.101e-06	4.246e-07	9.659	<2e-16	***
**TRAFNEAR**	3.209e-04	9.890e-06	32.444	<2e-16	***
**INDUS_5000**	1.525e-07	3.481e-08	4.381	1.19e-05	***

**NATUR_5000**	-8.172e-09	1.574e-08	-0.519	0.603678	
**RDL_500**	2.094e-04	3.433e-05	6.100	1.09e-09	***
**AGRI_300**	7.796e-06	2.785e-06	2.799	0.005138	**
**MRDL_300**	4.554e-04	1.349e-04	3.376	0.000737	***
**AIR_5000**	1.051e-07	3.976e-08	2.643	0.008239	**
**URBG_1000**	1.409e-06	2.052e-07	6.867	6.86e-12	***
**MRDL_50**	2.475e-02	1.240e-03	19.956	<2e-16	***
**Multiple R-squared: 0.3461,**		**Adjusted R-squared: 0.3454**			

Signif. codes: 0 '***' 0.001 '**' 0.01 '*' 0.05 '.' 0.1 ' ' 1

Table S5: SLR trained in Amsterdam

	Estimate	*Std. Error*	*t value*	*Pr(>|t|)*	
**(Intercept)**	6.906e+00	2.244e-01	30.780	<2e-16	***
**MRDL_100**	5.908e-03	1.533e-04	38.532	<2e-16	***
**TRAFNEAR**	1.069e-04	1.425e-06	75.012	<2e-16	***
**MRDL_25**	2.742e-02	9.877e-04	27.767	<2e-16	***
**PORT_1000**	9.889e-07	5.961e-08	16.590	<2e-16	***
**TRANS_5000**	1.048e-06	4.653e-08	22.523	<2e-16	***
**TLOA_1000**	4.916e-09	1.363e-10	36.059	<2e-16	***
**URBG_5000**	-1.209e-08	1.207e-08	-1.001	0.317	
**AGRI_300**	6.586e-06	6.140e-07	10.726	<2e-16	***
**INDUS_5000**	1.552e-07	1.601e-08	9.693	<2e-16	***
**WATER_1000**	1.404e-06	8.749e-08	16.048	<2e-16	***
**POP_1000**	3.543e-05	1.848e-06	19.176	<2e-16	***
**AIR_5000**	-9.398e-08	1.349e-08	-6.966	3.31e-12	***
**WATER_5000**	-9.105e-08	7.067e-09	-12.885	<2e-16	***
**NATUR_5000**	-6.473e-07	3.644e-08	-17.765	<2e-16	***
**Multiple R-squared: 0.4427,**		**Adjusted R-squared: 0.4425**			

Signif. codes: 0 '***' 0.001 '**' 0.01 '*' 0.05 '.' 0.1 ' ' 1

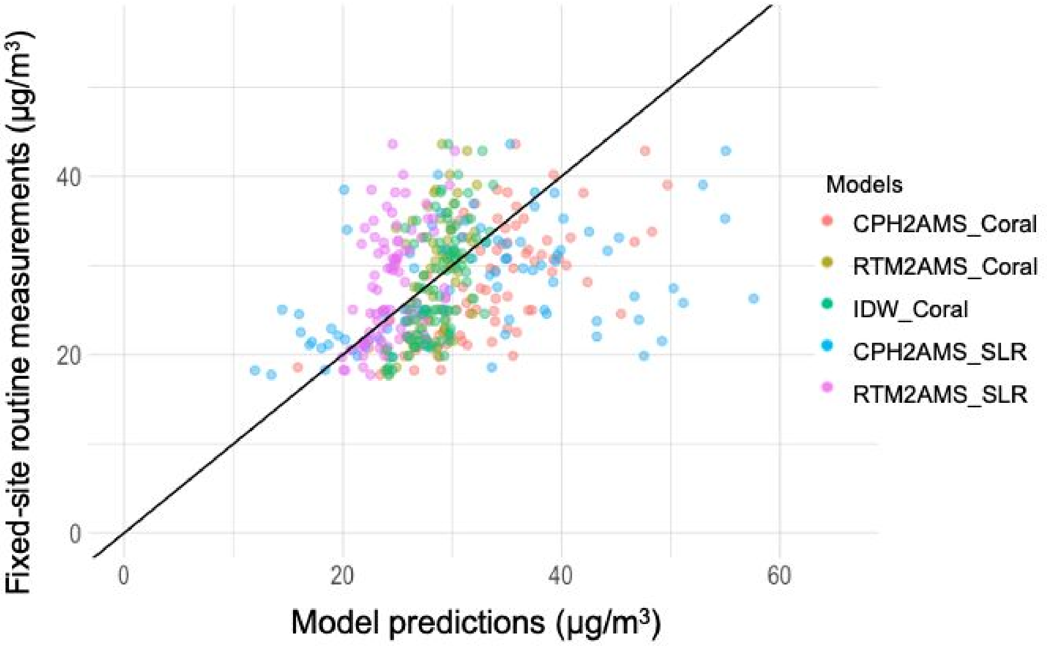

Figure S1. Scatterplot of model predictions against fixed-site validation measurements (NO_2; n=82).

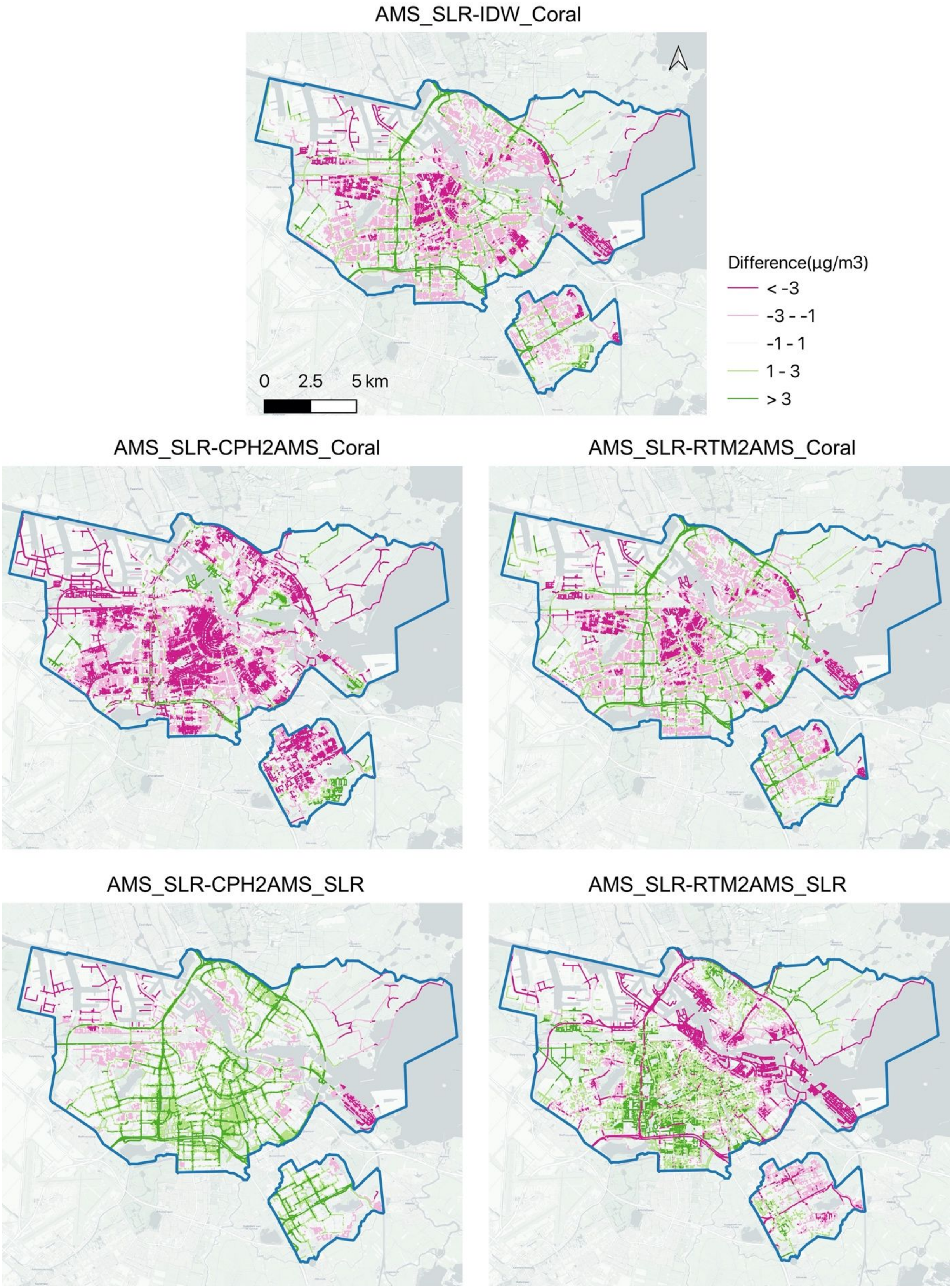

Figure S2. NO_2 maps of differences between SLR_AMS_160D and the other model tested.

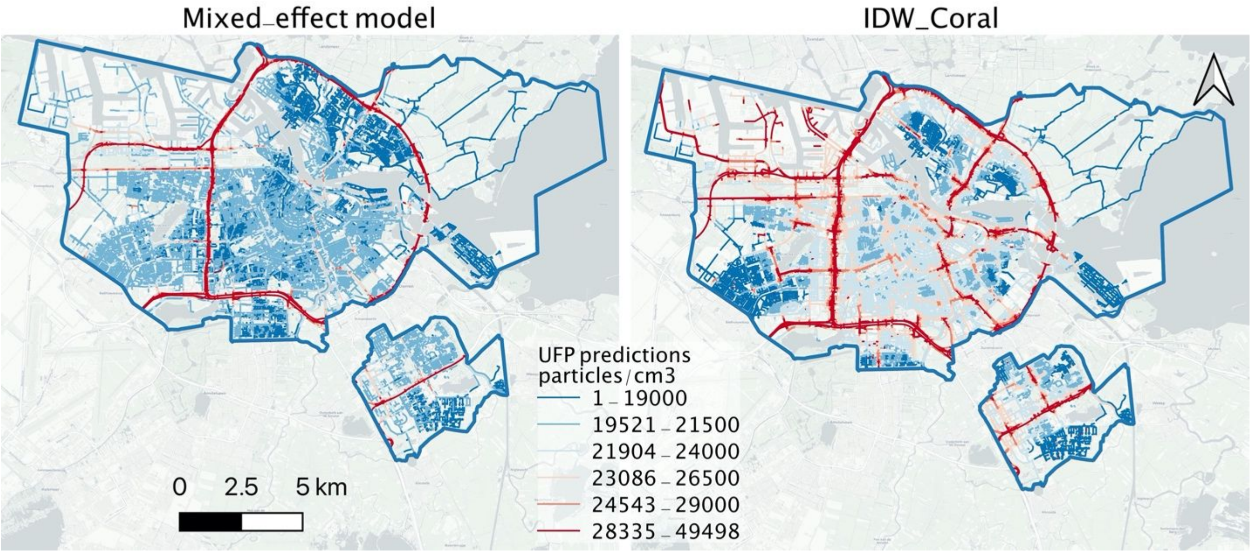

Figure S3. Spatial maps of UFP predictions from the mixed-effect and IDW_Coral models with a unified legend.
